# Dysphagia of aberrant right subclavian artery treated by endoscopic dilation: An alternative to surgical treatment in select cases—A case report

**DOI:** 10.1016/j.ijscr.2019.11.033

**Published:** 2019-11-27

**Authors:** Homa Sadeghian, Troy A. Moritz

**Affiliations:** a Department of Surgery, UPMC Pinnacle Harrisburg, Harrisburg, PA, USA; b Department of Cardiothoracic Surgery, UPMC Pinnacle Harrisburg, Harrisburg, PA, USA

**Keywords:** Aberrant subclavian artery, Dysphagia, Treatment, Endoscopy, Dilation, Case report

## Abstract

**Introduction:**

The aberrant right subclavian artery (ARSA) is a rare cause of dysphagia. Surgical intervention has remained the mainstem of therapy, accompanied with certain morbidities and mortalities. Although rarely reported in literature, endoscopic dilation may be considered a suitable treatment alternative in patients who are not a surgical candidate or do not consent for surgery. We report a case suffering from dysphagia and diagnosed with ARSA treated by endoscopic dilation.

**Presentation of case:**

A 52-year-old male presented to our clinic in 2015 with dysphagia. Chest Computed Tomography scan confirmed the diagnosis of ARSA. He first underwent esophagogastroduodenoscopy (EGD) with staged dilation of the stricture, making him free of his symptoms for an approximate 2.5 years. Upon recurrence of symptoms in 2018, he underwent repeat endoscopic dilation, which again completely resolved the symptom with an excellent peri-operative and post-operative course.

**Conclusion:**

Endoscopic dilation of the esophageal stricture in patients with ARSA is a safe alternative to surgery in patients who are unable or unwilling to undergo surgery. It provides relief for a relatively long time and can be safely repeated multiple times upon recurrence.

## Highlights

• Aberrant right subclavian artery (ARSA) is a rare cause of dysphagia.
• Surgical intervention is the mainstem of therapy.
• Endoscopic dilation is a suitable treatment alternative in non-surgical candidates.

## 1 Introduction

The aberrant right subclavian artery (ARSA) is a rare anatomical variation with a reported prevalence of 0.4% in the general population and 0.2–2.5% in autopsy series [1,2]. Due to its course behind esophagus, it may be a rare cause of dysphagia in patients (also called ‘dysphagia lusoria’) [3]. Diagnosis can be made through chest computed tomography (CT), chest CT angiography (CTA) or magnetic resonance angiography (MRA) [4].

Based on the rarity of the condition, there are no treatment guidelines. Although surgical intervention has remained the mainstem of therapy [1], the best surgical approach also remains controversial [5]. Different approaches include hybrid vascular and endovascular repair [4], and direct surgical repair. The latter can be obtained through a variety of corridors including extrathoracic [[5], [6], [7], [8]], or transthoracic (thoracotomy) [9,10], all accompanied with certain morbidities and mortalities.

Although endoscopy has been proposed helpful in the diagnosis of the condition [2,11,12], there is only a single report of its implication in the treatment of dysphagia caused by ARSA [13]. Given its more widespread availability, ease of performance, lower complication and morbidity profile, and lower cost compared to surgical repair, endoscopic dilation may be considered a suitable treatment alternative in patients who are not a surgical candidate or do not consent for surgery.

Here, we present a case presented to a community teaching hospital, suffering from dysphagia and diagnosed with ARSA treated by endoscopic dilation, and report his course over 3 years. To our knowledge, it is only the second report of the use of endoscopic dilation in this condition, and the first one of its implication in recurrent dysphagia due to ARSA.

The work has been reported in line with the Surgical Case Reports (SCARE) [14].

## 2 Presentation of case

**Fig. 1:**
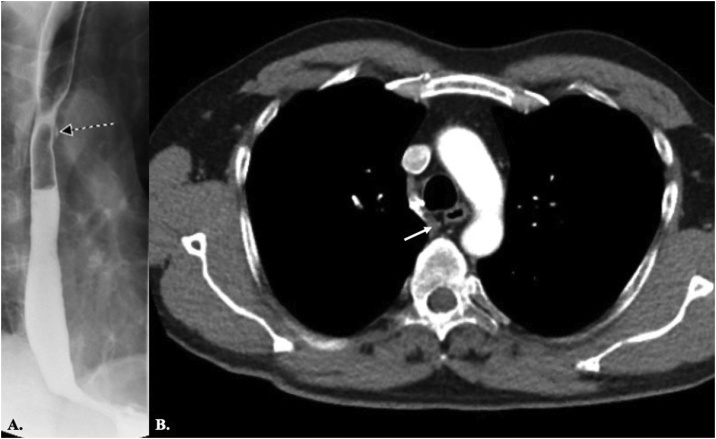
A) The barium swallow study shows an external compression over the esophagus (black arrow). B) Chest CT before treatment shows vascular ring and aberrant right subclavian artery with mass effect over and deviation of the esophagus (white arrow).

The patient was a 52-year-old former smoker right-handed white male, who had initially presented to our outpatient clinic in 2015 with dysphagia and difficulty in swallowing. Past medical history was positive for hypertension, hyperlipidemia, hepatic steatosis, chronic renal disease, history of nephrolithiasis, and allergy to Cephalosporins, Penicillin, and sulfonamide antibiotics. Physical exam was insignificant and Body Mass Index (BMI) and lab data were within normal limits. Barium swallow study showed an external compression over esophagus (Fig. 1a). Chest CT with contrast scan finally confirmed the diagnosis of ARSA (Fig. 1b). After a thorough discussion about treatment options, he declined any type of surgical intervention for correction of the anomaly. Therefore, a decision was then made to undergo esophagogastroduodenoscopy (EGD) with staged dilation of the stricture. In December 2015, the procedure was done successfully and the stricture site dilated to 38 F. The procedure was only complicated by a small mucosal tear which was managed conservatively and the patient left the hospital without sequela. His symptoms reduced after the dilation but did not completely resolve due to severity of the stricture. Therefore, few months later in February 2016, the second stage of dilation was repeated and 51 F of dilation obtained. He was symptom-free after the staged dilation for an approximate 2.5 years. However, he started to have slow progress of the same symptoms (dysphagia and swallowing difficulty) in August 2018, for which he presented to clinic in September 2018. Chest CT was repeated to rule out changes in anomaly, including formation of aneurysm in the aberrant artery. The CT again confirmed retroesophageal ARSA with common origin of the common carotid arteries. After a thorough discussion, he was consented for endoscopic dilation again.

### 2.1 Endoscopic procedure

**Fig. 2:**
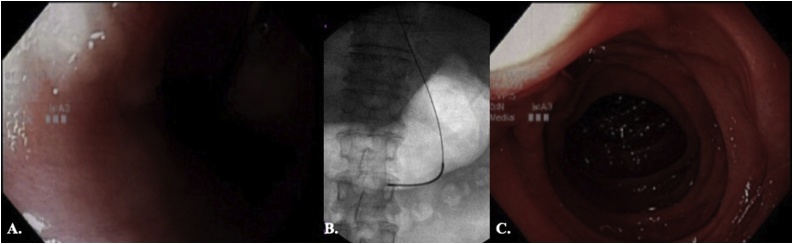
A) Esophagoscopy showed the stricture with normal mucosa. B) under fluoroscopy view, a guide wire was inserted for the dilator and the endoscope was withdrawn. C) esophagoscopy after dilation shows resolution of stricture.

The patient was intubated and prepared and draped in a standard manner. A standard EGD scope (Evis Exera III, Olympus, Olympus Corporation of the Americas, Center Valley, PA) was inserted. Mucosa appeared normal. Upon advancement of EGD scope, a short and shallow stricture was observed at the middle third of esophagus (Fig. 2a). At this point, we advanced the guide wire under fluoroscopy and withdrew the scope, leaving the guide wire in place (Fig. 2b). We serially dilated the stricture area over the guide wire under fluoroscopic view. Endoscopic Balloon Dilators (EZDilate, Olympus, Olympus Corporation of the Americas, Center Valley, PA) with increasing sizes (27, 30, 33, 36, 38 French) were used sequentially. After the favorable amount of dilation was obtained, we passed the scope through the previous stricture site and completed the EGD (Fig. 2c). The scope was then withdrawn. The procedure was performed by the main author (TAM, attending cardiothoracic surgeon).

No complications happened during EGD and dilation and the post-procedure chest XR showed no acute abnormalities. The patient tolerated the procedure well and left the hospital the same day without complications. During his first visit after the procedure, he was free of his symptoms and had no procedure-related complications.

## 3 Discussion

Aberrant right subclavian artery (ARSA) is a congenital anomalous course of the subclavian artery, rising directly from the aortic arch and crossing posterior to the esophagus. Different prevalence rates have been reported based on the study type, ranging from 0.2 to 13.3% in cadaveric studies and 0.4% in imaging studies [2,3]. Due to its retroesophageal course, it sometimes manifests as dysphagia. Historically, the main treatment protocol in symptomatic cases has been surgical repair. Different authors have proposed different surgical approaches. The most common surgical approaches include right supraclavicular extrathoracic approach, sternotomy/thoracotomy followed by transmediastinal approach, and combined use of endovascular and open vascular approaches, each with its own advantages and disadvantages [1,[4], [5], [6], [7], [8]].

In this report, we presented a patient with ARSA initially referred to us 3 years ago. The patient had refused any surgical treatment by then. Therefore, we decided to undergo endoscopic dilation to give him symptomatic relief. Although endoscopy has been reported of diagnostic value in ARSA [3], there is rarity of reports regarding its therapeutic effects. Till date, there is only one report regarding endoscopic dilation in ARSA-induced dysphagia. Bogliolo et al. reported a patient with ARSA treated by endoscopic esophageal dilation. In their case report, dilation was gained through sequential use of Celestin’s Neoplex dilators with increasing sizes (18–54 French) without complications [13]. We obtained dilation in our patient for the first time to a 51 F, after which he experienced relief for almost 2.5 years. Upon recurrence of dysphagia and based on his continued refusal of surgery, we repeated the endoscopic dilation to 38 F, with excellent results.

These 2 experiences show that even though endoscopic dilation only provides palliative relief of dysphagia (in contrast to the surgical repair which offers permanent cure) and there is a considerable probability of recurrence, it still can be considered as a treatment option due to its significantly lower morbidity, mortality, and cost and shorter hospital course. Our experience indicates that it can be considered a suitable treatment in select cases in whom surgery is not anticipated because of high risk profile or when the patient does not consent for surgery. Another potential minimally invasive therapeutic option that can be gained through endoscopy is insertion of an esophageal prosthesis. While widely used in esophageal stricture as a result of a variety of conditions (malignancy, lower esophageal spasm, etc.), endoscopic prostheses have not been used in stricture due to ARSA yet. Therefore, we decided to follow a less invasive treatment in our patient first. Insertion of a prosthesis is a viable choice that could be kept in mind by physicians in this rare condition.

The major limitation of this report is that it is based on a single case, making definite and strong recommendations impossible. More studies recruiting a higher volume of patients are needed to make a better conclusion.

## 4 Conclusion

Our limited report indicates that endoscopic dilatation is a minimally invasive treatment modality that can be considered in select ARSA cases in whom surgery is not anticipated because of high risk profile or when the patient does not consent for surgery.

## Sources of funding

None.

## Ethical approval

UPMC Pinnacle Harrisburg ethics committee.

## Consent

Written informed consent was obtained from the patient for publication of this case report and accompanying images. A copy of the written consent is available for review by the Editor-in-Chief of this journal on request.

## Author contribution

Homa Sadeghian collected patient’s data, drafted the manuscript and critically revised the final format. Dheera Rheedy critically revised the final format. Troy Moritz planned and performed the procedure and critically revised the final format of the manuscript.

## Registration of research studies

N/A.

## Guarantor

Dr. Troy Moritz.

## Provenance and peer review

Not commissioned, externally peer-reviewed.

## Declaration of Competing Interest

None.
